# Antigenotoxic properties of *Paliurus spina-christi* Mill fruits and their active compounds

**DOI:** 10.1186/s12906-017-1732-1

**Published:** 2017-04-26

**Authors:** Murat Zor, Sevtap Aydin, Nadide Deniz Güner, Nurşen Başaran, Arif Ahmet Başaran

**Affiliations:** 1 Department of Pharmacognosy, Faculty of Pharmacy, Bülent Ecevit University, 67100 Zonguldak, Turkey; 2 Department of Pharmaceutical Toxicology, Faculty of Pharmacy, Hacettepe University, 06100 Ankara, Turkey; 3 Republic of Turkey Social Security Institution, 06520 Ankara, Turkey; 4 Department of Pharmacognosy, Faculty of Pharmacy, Hacettepe University, 06100 Ankara, Turkey

**Keywords:** *Paliurus spina-christi* Mill, Active compounds, Homo- and hetero-NMR, MTT assay, Comet assay, Chinese hamster lung fibroblast cells

## Abstract

**Background:**

*Paliurus spina-christi Mill.* (PS) fruits are widely used for different medical purposes in Turkey. Like in many medicinal herbs the studies concerning their activity, the activities of PS are also not well clarified. The aim of this study is to evaluate the antigenotoxicity of the compounds isolated and identified from the extracts of PS fruits.

**Methods:**

The active compounds were separated, isolated, and determined by chromatographic methods and their structural elucidation was performed by Nuclear Magnetic Resonance (NMR) methods. The compounds were obtained from either ethyl acetate (EA) or n-butanol extracts. The cytotoxicities of the compounds using 3-(4,5-dimethylthiazol-2-yl)-2,5-diphenyltetrazolium bromide (MTT) assay and the antigenotoxic activities of the compounds using the alkaline single cell gel electrophoresis techniques (comet assay) were evaluated in Chinese hamster lung fibroblast (V79) cell lines.

**Results:**

The isolated major compounds were identified as (+/−) catechins and gallocatechin from EA fraction and rutin from n-butanol fraction of PS fruits. Their chemical structures were identified by ^1^H-NMR, ^13^C-NMR, HMBC, and HMQC techniques. Half-maximal inhibitory concentration of catechins, gallocatechin, and rutin were found to be 734 μg/mL, 220 μg/mL, and 1004 μg/mL, respectively. The methanolic extract of PS (1-100 μg/mL) alone did not induce DNA single-strand breaks while catechins (1-100 μg/mL), gallocatechin (1-50 μg/mL), and rutin (1-50 μg/mL) significantly reduced H_2_O_2_-induced DNA damage.

**Conclusion:**

It has been suggested that PS fruits and their compounds catechins, gallocatechin and rutin may have beneficial effects in oxidative DNA damage. It seems that PS fruits may be used in protection of the disorders related to DNA damage.

## Background

Medical treatments with herbs in Turkey having rich resources in terms of folk remedies are widely used. The arising interest of using natural compounds for health purposes has focused attention on plants having rich sources of bionutrient or bioactive phytochemicals. Many traditional usage of herbs are reviewed and their extracts or compounds are used in the treatment of some diseases such as cancer, cardiovascular disorders, diabetes, and sepsis [1, 2]. Reactive oxygen species (ROS) has an important role in the aetiology of several diseases. In general, phenolics are accepted as potentially scavengers of free radicals and their antioxidant capacities may vary due to their different chemical structures. The well known biological activity of catechins is their antioxidant and free radical scavenging properties. Catechins are dietary polyphenolic compounds associated with a wide variety of beneficial health effects in in vitro and in various animal models [3, 4]. On the other hand, it is important to remember that under certain conditions, the phenolic structures, especially flavonoids, may undergo auto-oxidation and act as pro-oxidants as well [5].

*Paliurus spina-christi* Mill. (PS) (Rhamnaceae), also known as Christs thorn or Jerusalem thorn is found widely in Mediterranean region. PS fruits contain tannins, alkaloids, sterols, flavonoids such as rutin, isoquercetin, quercetin-3-rutinoside-7-rhamnoside, kaempferol-3-glycoside and epigallocatechol, gallocatechol, catechol [6, 7]. In our early study, quercetin, rutin, isoquercitrin, gallocatechin, (+) and (−) catechin, quercetin-3-*O*-α-rhamnosyl(1 → 6)-β-galactoside, quercetin-3-*O*-[−β-xylosyl(1 → 2)-α-rhamnozil(1 → 6)]-β-glucoside, quercetin-3-*O*-α-rhamnosyl(1 → 6)-β-galactoside-7-*O*-α-rhamnoside were isolated from dried PS fruits [8].

PS is traditionally used as diuretic, antirheumatic, hypocholesterolemic and tonic as well as in inflammation, diarrhea, and chronic obstructive pulmonary disease [7]. Its water extract and their compounds show antimicrobial, antibacterial, antifungal, hypolipidemic and antioxidant activities [9–11]. Tsarubol was isolated from the whole fruit of PS and its therapeutic efficacy on chronic hepatocholecystitis was demonstrated by Kemertelidze et al. [12]. To continue our studies on the evaluation of the protective effects of the medicinal plants on genotoxicity by comet assay [13–15], we decided to examine the effects of PS extract also. Being a sensitive, rapid, simple and multidirectional method, comet assay is getting popular in determining antigenotoxicity of herbal extracts in vitro and also known as single cell gel electrophoresis assay. The method was found by Ostling and Johanson [16] developed by Singh et al. [17], and used for the measuring DNA strand breaks in eukaryotic cells at single cell level [18, 19]. The previous in vitro and in vivo studies, showed the protective effects of some phenolic compounds on oxidative DNA damage by comet assay [20–33].

With this study, the chemical structures of the major active compounds of PS fruits were elucidated by NMR methods and named as catechins, gallocatechin and rutin. Their cytotoxic effects were determined in Chinese hamster lung fibroblast (V79) cell lines. Then, the protective effects of the compounds against H_2_O_2_-induced DNA damage were investigated by comet assay in vitro.

## Methods

### Materials

The chemicals used in the experiments were purchased from the following suppliers: Fetal calf serum (FCS), trypsin–EDTA, RPMI 1640, penicillin-streptomycin, L-glutamine from Biological Industries (Kibbutz Beit-Haemek, Israel); low melting point agarose (LMA), 3-(4,5-dimethylthiazol-2-yl)-2,5-diphenyltetrazolium bromide (MTT) dye, methylene blue dye, and normal melting point agarose (NMA) from Boehringer Mannheim (Mannheim, Germany); acetic acid, acetone, n-butanol, n-butanon, EA, formic acid, n-hekzan, chloroform (CHCl_3_), methanol (MEOH), sodium chloride (NaCl), sodium hydroxide (NaOH), sulphuric acid, toluen from Merck Chemicals (Darmstadt, Germany); dimethyl sulfoxide (DMSO), ethanol, ethidium bromide (EtBr), methanol, phosphate buffered saline (PBS) from Sigma (St. Louis, MO, USA); ethylenediamine tetra acetic acid disodium salt dihydrate (Na_2_-EDTA), N-lauroyl sarcosinate, tris, and Triton X-100 from ICN Biomedicals Inc. (Aurora, Ohio, USA). Water (H_2_O) was supplied from in a Milli-Q (Millipore, Bedford, MA, USA) water purification system.

### Preparation of plant extracts

*Paliurus spina-christi* Mill. (PS) was identified by the Department of Pharmaceutical Botany of Hacettepe University and the voucher specimens (HUEF 99-078 and HUEF14-078) were deposited at the herbarium of the Faculty of Pharmacy, Hacettepe University, Turkey.

PS fruits were collected from Kastamonu region of Turkey in July 2014 and kept in air-dried room temperature (20 ± 2 °C). Air dried fruits (300 g) were powdered using a mechanical grinder and extracted with 1000 mL of MeOH:H_2_O (70:30 *v*/v) in 40 °C in a continuous extractor for 48 h. The extract was then concentrated at 40 °C using a rotary evaporator (Büchi RE 111, Büchi EL 131, Flawil, Switzerland) and used for the phytochemical investigations. The extract was pre-fractionated with n-hexane, EA, and n-butanol, respectively.

### Phytochemical studies of the PS extracts

The EA fraction (1.1 g) and the n-butanol fraction (8.5 g) were first investigated by TLC (thin layer chromatography). The fractions rich in phenolics were applied to a polyamide chromatography separately using a gradient system of MeOH and H_2_O from 0 to100. Following the fractions rich in phenolics were applied to column chromatography method using silicagel (Kieselgel 60,70-230 mesh) with the elution system CHCl_3_:MeOH (90:10 to 25:75 *v*/v). The lyophilization of these fractions was performed by a lyophilizer (VirTis Freezemobile 6, SP Scientific, NY, USA). The isolation of (−) catechin (2.33 mg) from EA fraction were carried out in Sephadex LH20 column using MeOH. A fraction from the same sephadex LH20 column was first applied to silicagel (Kieselgel 60,70-230 mesh) column with the elution system EA:Formic acid:Acetic acid:H_2_O (100:11:11:27). Gallocatechin (5.63 mg) and (+) catechin (3.61 mg) were isolated and purified by preparative TLC (Kieselgel 60) using the solvent system CHCl_3_:MeOH:H_2_O (61:32:7). Rutin was isolated from polyamide column after elution with methanol:water (50:50) and purified by precipitation. The structure elucidations of the compounds were performed by homo and hetero Nuclear Magnetic Resonance (NMR) techniques (^1^H-NMR 600 MHz, CD_3_OD; ^13^C-NMR 150 MHz, CD_3_OD: Jeol Ecp-600 FT-NMR, MA USA).

The concentrations of the compounds were increased using the same systems to use in biological experiments. (+) and (−) Catechins were used together in the experiments.

### Cell culture

V79 cells were purchased from American Type Culture Collection (Manassas, VA, USA). The cells were maintained and grown as a monolayer in culture plastic flasks (75 cm^2^). The culture medium was RPMI 1640 medium with L-glutamine, containing 10% heat-inactivated fetal bovine serum, 100 U/L penicillin, and 100 μg/mL streptomycin. The cells were kept in a incubator (Thermo Scientific Heraeus, Germany) at 37 ± 1 °C with a humidified atmosphere of 95% air and 5% CO_2_. The culture medium was renewed every 3 days. V79 cells used in all experiments were between the 3rd and 5th culture passage after thawing.

### Determination of cytotoxicity

The proliferative or cytotoxic effects of PS extracts were determined using MTT assay, first described by Mosmann [34] with the modification of Denizot and Lang [35]. V79 cell line was chosen because of its high sensitivity to various chemicals with excellent properties in colony formation. These adherent cells are very convenient practically in MTT assay. After growing for 2 weeks, V79 cells were seeded at a density of 5000 cells/well in a 96-well plate and allowed to grow for 24 h before treatment. The cells were treated with each compounds at different concentrations (0, 1, 5, 10, 25, 50, 100, 250, 500, 1000, and 2000 μg/mL) in the culture medium for 24 h. The compounds were dissolved in DMSO and added to the medium to yield a final DMSO concentration of 0.5% (*v*/v). Control experiments were carried out with the culture medium containing 0.5% of DMSO without PS extracts. Later the medium was removed and 0.5 mg/mL MTT solution in 100 μL of the culture medium was added to each well and further incubated at 37 °*C. medium* was discarded after 4 h, and 100 μL of PBS was added to wash the cells. After removing PBS, 100 μL of DMSO was added to dissolve the formazan crystals at 37 °C for 10 min. Absorbance of each sample was measured at 570 nm using the microplate reader (SpectraMax M2, Molecular Devices Limited, Berkshire, UK). Cytotoxicity was determined by the percentage of the ratio between treated and untreated (control) cells (% cell viability) using Eq. (1). A_blank_ and A_sample_/_control_ indicate the absorbance of blank and absorbances of samples or control, respectively. IC_50_ values of the compounds, the concentration reducing the cell viability of treated cells by 50% with reference to the control (untreated cells), were determined from the dose-response curves. Four independent assays were performed.

1 $$ \mathrm{Percentage}\ \mathrm{of}\ \mathrm{cell}\ \mathrm{viability}\ \left(\%\mathrm{cell}\ \mathrm{viability}\right)=\left({\mathrm{A}}_{\mathrm{samples}}\hbox{--} {\mathrm{A}}_{\mathrm{blank}}\right)/\left({\mathrm{A}}_{\mathrm{control}}\hbox{--} {\mathrm{A}}_{\mathrm{blank}}\right)\times 100 $$

### Determination of genotoxicity and antigenotoxicity

We determined the genotoxic or antigenotoxic effects of the samples at the concentration of less than IC_50_ by alkaline comet assay in V79 cells. The alkaline comet assay was carried out to analyze DNA single strand breaks as described by Tice et al. [36] with minor modifications. V79 cells were harvested by treatment with 0.15% trypsin-0.18% EDTA in the medium without FBS. Then the cells were seeded (25 × 10^3^ cells) in 2 mL of the culture medium in a six-well plate and grown for 24 h up to 60-70% confluence before treatment with the compounds in the incubator. The cells were incubated with the compounds at non-cytotoxic concentrations (1, 5, 25, 50, and 100 μg/mL) in the culture medium for 1 h. Final DMSO concentration in the medium never exceeded 0.5%. After pretreatment of the compounds for 1 h, oxidative DNA damage was induced by replacing the medium with PBS containing 100 μM H_2_O_2_ and then the cells were incubating for 5 min to assess the antigenotoxicity of the compounds. Then the cells were harvested by treatment with 0.15% trypsin-0.18% EDTA in the medium without FBS, centrifuged, and washed with PBS for removing the H_2_O_2_ solution. A negative control (0.5% DMSO) and positive control 100 μM H_2_O_2_ were also included in the experiment. After centrifugation at 1100 rpm, supernatant was discarded. 1 × 10^4^ cells in 50 μL PBS were suspended in 100 μL of 0.65% LMA. Then the suspensions were embedded on the slides pre-coated with a layer of 1% NMA. The slides were allowed to solidify on ice for 5 min. Coverslips were then removed. The slides were immersed in cold lysing solution (2.5 M NaCl,100 mM EDTA, 100 mM Tris, 1% sodium sarcosinate, pH 10.0), with 1% Triton X-100 and 10% DMSO added just before use for 3 h at 4 °C. Then they were removed from the lysing solution, drained and were left in the electrophoresis solution (1 mM sodium EDTA and 300 mM NaOH, pH 13.0) for 20 min at 4 °C to allow for unwinding of the DNA and expression of alkali-labile damage. Electrophoresis was conducted also at a low temperature (4 °C) for 20 min using 25 V with a current of 300 mA. The slides were neutralized by washing 3 times in 0.4 M Tris-HCl (pH 7.5) for 5 min at room temperature. After neutralization, the slides were incubated in 50%, 75%, and 99% of alcohol for 5 min, successively. The dried microscope slides were stained with EtBr (20 μg/mL in distilled H_2_O, 35 μL/slide), covered with a cover-glass prior to analysis with a fluorescence microscope (Leica Microsystems GmbH, Wetzlar, Germany) under green light. The microscope was connected to a charge-coupled device camera and a personal computer-based analysis system (Comet Analysis Software, version 3.0, Kinetic Imaging Ltd., Liverpool, UK) to determine the extent of DNA damage after electrophoretic migration of the DNA fragments in the agarose gel. In order to visualize DNA damage, slides were examined at 40×. The experiment was repeated four times. One-hundred cells from two replicate slides were assayed for each experiment. DNA damage was expressed as the product of the tail length and the fraction of total DNA in the tail “DNA tail moment”.

### Statistical analysis

The statistical analysis was performed using the software programs SPSS 15.0 (SPSS, Chicago, IL, USA). The distribution of the data was checked for normality using the Shapiro-Wilk test. The homogeneity of the variance was verified by the Levene test. Differences between the means of data were compared by the one way variance analysis test and post hoc analysis of group differences was performed by least significant difference test. The results were given as the mean ± standard deviation. *P* value of less than 0.05 was considered as statistically significant.

## Results and discussion

### Determination of phytochemical constituents of PS extracts

**Fig. 1:**
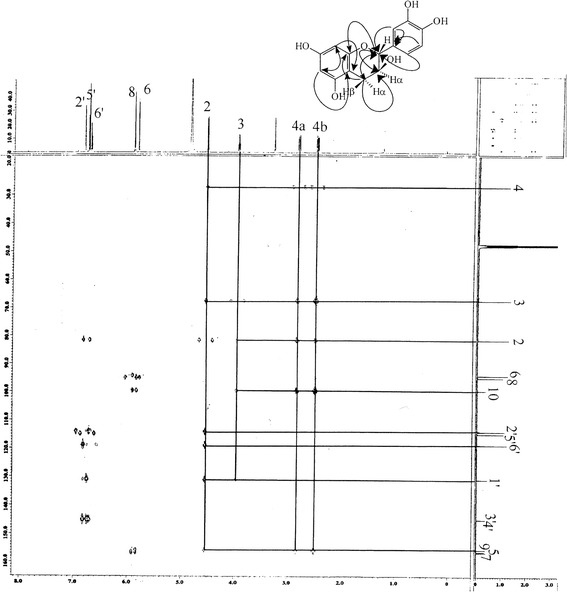
HMBC spectrums of (+, −) catechin

**Fig. 2:**
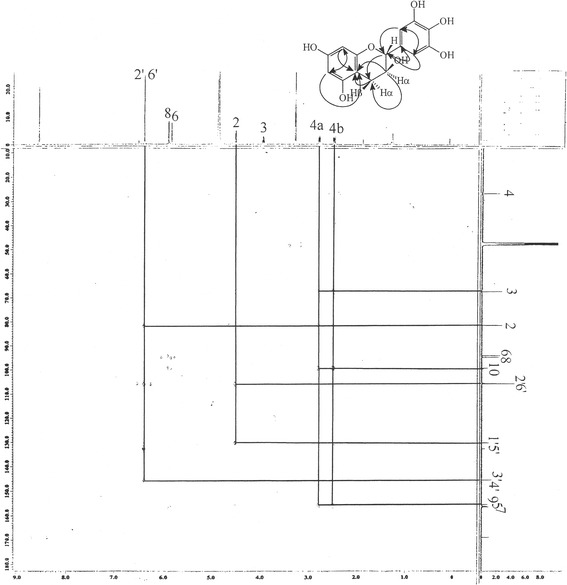
HMBC spectrums of (−)-gallocatechin

The compounds (+), (−) catechins and gallocatechin were isolated from EA fraction by chromatographic methods as done previously [8]. The chemical elucidation of the isolated compounds was identified by ^1^H- and ^13^C-NMR method (Tables 1 and 2). The interpretation of the proton signals was realized by the spectrum of 2D-^1^H -^1^H homo and heteronuclear correlation (COSY) and the shared spin systems show the presence of the protons of phenyl ring with other protons, where the results were in good accordance with references. Carbon signals with short distance proton signals, methylene signals, 2 non substitute methine groups and other signals supported both catechin and gallocatechin skeleton in the HMQC spectrum. HMBC spectrum of gallocatechin showed interactions with the protons especially the long distance interactions of H-2 proton with C-1′ and protons of H-3; H-4; H-6 and H-8 with C-10 confirms the skeleton (Fig. 1). The confirmation of the catechin structure obtained from the long term interactions of protons H-2 and H-5 with C-1′ and H-3, H-4, H-6, and H-8 with C-10 with the correlated NMR techniques was also shown in Fig. 2. The major compound of n-butanol fraction was identified as rutin, a flavonoid glycoside, from the data obtained by ^1^H- and ^13^C-NMR analysis showing the chemical structure clearly (Table 3).

**Table 1:** Spectral determination of (+/−) catechins in ^1^H-NMR and ^13^C-NMR^a^

Atom C/H	δ_H_ (ppm)	J (Hz)	δ_C_ (ppm)	HMBC (H → C)
2	4.55 d	7.4	82.88	C-4, C-3, C-9
3	3.96 ddd	5.5/7.5/8.1	68.83	C-10
4	(*α*) 2.84 dd	15.9/5.5	28.54	C-2, C-3, C-9, C-10
	(*β*) 2.84 dd	15.9/8.0		
5	-	-	156.92	
6	5.84 d	2.2	95.51	C-8, C-10
7	-	-	157.59	
8	5.91 d	2.2	96.30	C-6, C-10
9	-	-	157.86	
10	-	-	100.83	
1′	-	-	132.35	
2′	6.82 d	1.9	115.27	C-1′, C-2
3’	-	-	146.23	
4’	-	-	146.23	
5’	6.75 d	8.2	116.08	C-1′
6’	6.70 dd	8.2/1.9	120.03	C-2

^a1^H-NMR:600 MHz, CD_3_OD; ^13^C-NMR:150 MHz, CD_3_OD

**Table 2:** Spectral determination of (−)-gallocatechin in ^1^H-NMR and ^13^C-NMR^a^

Atom C/H	δ_H_ (ppm)	J (Hz)	δ_C_ (ppm)	HMBC (H → C)
2	4.51 d	7.2	82.89	C-4, C-1’
3	3.95 m	-	68.78	C-4, C-10
4	(*α*) 2.80 dd	16.2/5.5	28.13	C-10
	(*β*) 2.48 dd	16.2/7.7		
5	-	-	156.85	
6	5.84 d	2.2	95.53	C-8, C-10
7	-	-	157.60	
8	5.91 d	2.2	96.28	C-6, C-10
9	-	-	157.84	
10	-	-	100.73	
1’	-	-	131.60	
2’	6.39 s	-	107.20	C-2, C-6′
3’	-	-	146.87	
4’	-	-	134.01	
5’	-	-	146.87	
6’	6.39 s	-	107.20	C-2, C-2’

^a1^H-NMR:600 MHz, CD_3_OD; ^13^C-NMR:150 MHz, CD_3_OD

**Table 3:** Spectral determination of rutin in ^1^H-NMR and ^13^C-NMR^a^

Atom C/H	δ_H_ (ppm)	J (Hz)	δ_C_ (ppm)
2	-	-	82.89
3	-	-	68.78
4	-	-	28.13
5	-	-	156.85
6	6.19 d	1.8	95.53
7	-	-	157.60
8	6.38 d	2.4	96.28
9	-	-	157.84
10	-	-	100.73
1’	-	-	131.60
2’	7.66 d	1.8	107.20
3’	-	-	146.87
4’	-	-	134.01
5’	6.86 d	8.5	146.87
6’	7.64 dd	8.5/1.8	107.20
β-Glucose			
1”	5.10 d	7.9	104.8
2”	3.46 dd	9.8/7.5	75.7
3”	3.25 t	9.8	78.1
4”	3.29 ϕ	ϕ	71.4
5”	3.38 *m*		77.1
6”a	3.79 dd	11.0/1.2	68.6
6”b	3.63 dd	12.0/4.9	
α-rhamnose			
1”	4.51 d	1.8	102.4
2”	3.63 dd	3.7/1.8	72.1
3”	3.53 dd	9.8/3.7	72.2
4”	3.28 t	9.8	73.9
5”	3.43 *m*	-	69.7
6”	1.12 d	6.7	17.9

^a1^H-NMR:600 MHz, CD_3_OD; ^13^C-NMR:150 MHz, CD_3_OD

ϕ: non calculated because of interference

The spectral data of the compounds were also confirmed as (+) (−) catechin, gallocatechin, and rutin by comparing with authentic samples and literature data [37–42].

### Cytotoxicity of catechins, gallocatechin, and rutin

**Fig. 3:**
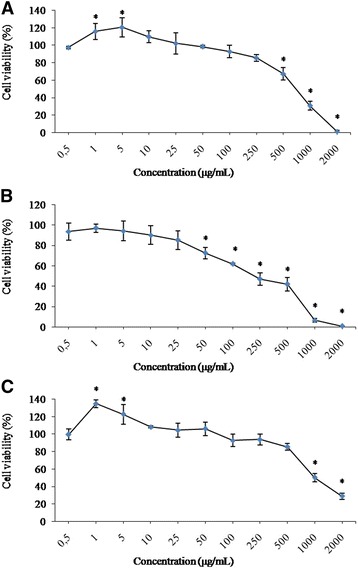
Cytotoxicity of (+/−) catechins (**a**), gallocatechin (**b**) and rutin (**c**) in V79 cells. ^*^ *p* < 0.05, compared with negative control (0.5% DMSO)

The cytotoxic effects of the compounds in wide concentration ranges in V79 cells were shown in Fig. 3a, b, and c. Catechins and rutin significantly increased the cell proliferation at the concentrations of 1 μg/mL and 5 μg/mL, respectively, for 24 h. The cell viabilities started to decline at the higher concentrations of 250 μg/mL, 25 μg/mL, and 500 μg/mL, respectively for catechins, gallocatechin and rutin. According to the results, catechins, gallocatechin, and rutin significantly decreased the cell viability at the concentrations of 500 μg/mL, 50 μg/mL, and 1000 μg/mL, respectively (*p* < 0.05). The concentration-dependent cytotoxicity was observed in V79 cells after 24 h exposure for catechins, gallocatechin, and rutin. The IC_50_ values of catechins, gallocatechin, and rutin in V79 cells were found to be 734 μg/mL, 220 μg/mL, and 1004 μg/mL, respectively. According to the IC_50_ values of the compounds, the rank order of cell growth inhibitory potency in V79 cells was arranged as gallocatechin> catechins>rutin.

Catechins significantly increased cell proliferation at the concentrations of 1 μg/mL and 5 μg/mL, resulting in 15.6% and 20.5% increase upon control, respectively (*p* < 0.05) (Fig. 3a). Rutin also increased the cell proliferation at the concentrations of 1 μg/mL and 5 μg/mL significantly, resulting in 34.7% and 22.6% upon control, respectively (*p* < 0.05) (Fig. 3c).

As there are limited studies on the toxicities of catechins, gallocatechin, and rutin, we examined the cytotoxic potencies of the compound as mentioned above in V79 cells. Our results showed that catechin, gallocatechin, and rutin significantly decreased the cell viability at the concentrations of 250 μg/mL, 50 μg/mL, and 500 μg/mL, respectively, in a concentration dependant manner.

Catechin was found as inhibitor in the growth of human liver carcinoma cell line (HepG2) cell lines at the concentrations of 100-200 μg/mL [43]. Significant decrease in cell viability was observed on V79 cells when treated with H_2_O_2_ (1 mM), while herbal extracts and its flavonoids and (−)-epigallocatechin-3-gallate (EGCG) prevented the lactate lactate dehydrogenase release from H_2_O_2_ cytotoxicity [44]. Total catechin content of green tea was reported to be 65.6 mg/g of dry matter and the green tea exhibited the lowest IC_50_ values (2 g fresh herb/100 mL) [44]. Cytotoxic effects of rutin for 48 h incubation in HL-60 leukemic cells were determined by MTT test. The IC_50_ values of rutin were about 14 mg/mL and the concentration-dependent cytotoxic effect of rutin was demonstrated [45]. In human leukaemia K562 cell lines (K562 cells), IC_50_ of rutin was found to be 400 μg/mL for 24 h and a dose dependant inhibition of cell viability was reported [46]. Rutin above the concentrations higher than 50 μg/mL exhibited a cytotoxic effect in HepG2 cells for 24 h treatment [47].

### Antigenotoxicity of catechins, gallocatechin, and rutin

**Fig. 4:**
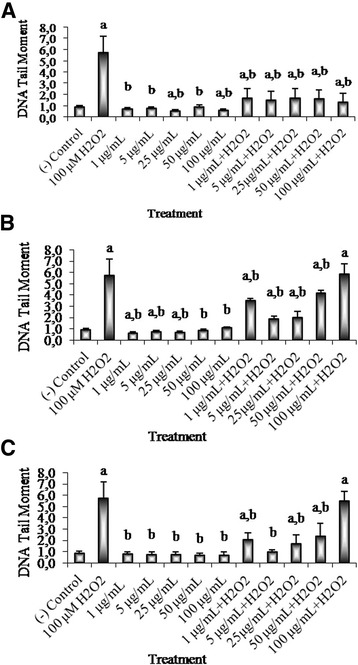
Gentoxicity/antigenotoxicity of (+/−) catechins (**a**), gallocatechin (**b**), and rutin (**c**). DNA damage was expressed as DNA tail moment. ^a^ *p* < 0.05, compared with negative control (0.5% DMSO); ^b^ *p* < 0.05, compared with 100 μM H_2_O_2_ (positive control)

**Fig. 5:**
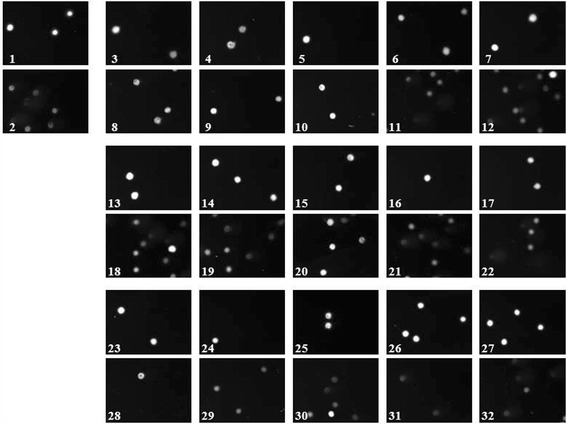
The images of cells for each treatment in comet assay. (1) Negative control (0.5% DMSO); (2) positive control (100 μM H_2_O_2_). (3) 1 μg/mL of catechins; (4) 5 μg/mL of catechins; (5) 25 μg/mL of catechins; (6) 50 μg/mL of catechins; (7) 100 μg/mL of catechins; (8) 1 μg/mL of catechins + H_2_O_2_; (9) 5 μg/mL of catechins + H_2_O_2_; (10) 25 μg/mL of catechins + H_2_O_2_; (11) 50 μg/mL of catechins + H_2_O_2_; (12) 100 μg/mL of catechins + H_2_O_2_; (13) 1 μg/mL of gallocatechin; (14) 5 μg/mL of gallocatechin; (15) 25 μg/mL of gallocatechin; (16) 50 μg/mL of gallocatechin; (17) 100 μg/mL of gallocatechin; (18) 1 μg/mL of gallocatechin + H_2_O_2_; (19) 5 μg/mL of gallocatechin + H_2_O_2_; (20) 25 μg/mL of gallocatechin + H_2_O_2_; (21) 50 μg/mL of gallocatechin + H_2_O_2_; (22) 100 μg/mL of gallocatechin + H_2_O_2_; (23) 1 μg/mL of rutin; (24) 5 μg/mL of rutin; (25) 25 μg/mL of rutin; (26) 50 μg/mL of rutin; (27) 100 μg/mL of rutin; (28) 1 μg/mL of rutin + H_2_O_2_; (29) 5 μg/mL of rutin + H_2_O_2_; (30) 25 μg/mL of rutin + H_2_O_2_; (31) 50 μg/mL of rutin + H_2_O_2_; (32) 100 μg/mL of rutin + H_2_O_2_

The results of the genotoxicity and antigenotoxicity of PS extracts in V79 cells using alkaline comet assay were shown in Figs. 4 and 5.

1-100 μg/mL of (+) and (−) catechin fraction significantly decreased H_2_O_2_-induced DNA damage (*p* < 0.05). A dose dependent protective effects in the concentrations of 1 μg/mL, 5 μg/mL, 25 μg/mL, 50 μg/mL, 100 μg/mL were observed (70.3%, 73.8%, 70.2%, 71.6%, and 76.5%, respectively, vs. positive control) (Fig. 4a).

Similarly, gallocatechin at the concentrations of 1-50 μg/mL was applied to the assay and at the concentrations of 1 μg/mL, 5 μg/mL, 25 μg/mL, and 50 μg/mL gallocatechin significant decreased oxidative DNA damage (39.2%, 67.3%, 65.5%, and 27.7%, for respectively, vs. positive control) (*p* < 0.05) (Fig. 4b).

The same dose dependent responses were observed in rutin and 1 μg/mL, 5 μg/mL, 25 μg/mL, and 50 μg/mL concentrations of rutin showed decreases in oxidative DNA damage (64.2%, 82.6%, 70.3%, 58.6% respectively, vs. positive control) (*p* < 0.05) (Fig. 4c).

In our study, we observed that catechins, gallocatechin, and rutin alone have no genotoxic effects below 100 μg/mL. Catechins at the concentrations between 1 μg/mL and 100 μg/mL and gallocatechin and rutin at the concentrations between 1 μg/mL and 50 μg/mL reduced DNA single-strand breaks induced by H_2_O_2_ in V79 cells, indicating that the compounds may have protective effect against oxidative DNA damage. It has been suggested that polyphenols suppress H_2_O_2_-induced DNA damage in the cells [48].

The results were in good correlation with the studies done before. The genotoxic and antigenotoxic activities of catechin in the bark of *Hamamelis virginiana* L. were investigated in HepG2 cells using comet assay to determine DNA damage. Catechin, hamamelitannin and two proanthocyanidin fractions of the plant extract were found causing slight DNA damage up to concentrations of 166 μg/mL. Pretreatment with catechin at a concentration of 18 μg/mL caused a 50% reduction of the benzo[a]piren induced genotoxicity [49]. Catechins have protective effects on oxidative DNA damage at low concentrations, while it has pro-oxidants effect at high doses. In human lymphocytes, 200 μM of EGCG increased oxidative DNA damage [3]. EGCG was also found to induce DNA double-strand breaks in human lung and skin cells above 30 μM [50]. In Jurkat T-lymphocytes, EGCG induced and inhibited oxidative DNA damage above 100 μM and 10 μM, respectively [51]. EGCG between 0.01-10 μM was found to decrease DNA damage induced by H_2_O_2_ in human lymphocytes [29].

In another study, it was reported that rutin pretreatment (50 μM) reduced the excessive reactive oxygen species and apoptosis, prevented the increased DNA fragment formation and glutathione depletion, and inhibited the collapse of mitochondrial membrane potentials in human umbilical vein endothelial cells exposed to H_2_O_2_ [52]. The induction of DNA damage in K562 cells after exposition to different concentrations of rutin for 24 h was studied using comet assay. Rutin alone did not cause significant DNA damage between the concentrations of 50-400 μg/mL and it decreased H_2_O_2_-induced DNA damage at the concentrations of 100, 200, and 400 μg/mL in K562 cells [46]. Also, at the concentrations of 0.1-200 μg/mL rutin did not induce genotoxicity in HepG2 cells for 2 h incubation [45].

## Conclusions

The compounds with high polarity were found in the methanolic extract of PS fruits by phytochemical survey. The isolated and identified compounds were (+) and (−) catechins and gallocatechin from ethyl acetate fraction and rutin from n-buthanol phase. The compounds were isolated and purified and elucidated by chromatographic and spectrophotometric methods.

The antigenotoxic studies using comet assay show that the isolated compounds from PS fruits may have protective effect against oxidative DNA damage, which support the ethnopharmacological properties and traditional uses of PS. It seems that PS fruit may be a good resource for clinical research on the treatment of the diseases related to oxidative DNA damage. However, further studies are needed to confirm the activity and clarify the mechanism of action of PS fruits.

## Abbreviations

CHCl_3_: Chloroform
COSY: 2D-H^1^-H^1^ homonuclear correlation
DMSO: Dimethyl sulfoxide
EA: Ethyl acetate
EGCG: (−)-Epigallocatechin-3-gallate
EtBr: Ethidium bromide
FCS: Fetal calf serum
H_2_O: Water
HepG2: Human liver carcinoma cell line
K562 cells: Human myeloid leukemia cell lines
LMA: Low melting point agarose
MEOH: Methanol
MTT: 3-(4,5-dimethylthiazol-2-yl)-2,5-diphenyltetrazolium bromide
Na_2_-EDTA: Ethylenediamine tetra acetic acid disodium salt dihydrate
NaCl: Sodium chloride
NaOH: Sodium hydroxide
NMA: Normal melting point agarose
NMR: Nuclear Magnetic Resonance
PBS: Phosphate buffered saline
PS: *Paliurus spina-christi* Mill.fruits
ROS: Reactive oxygen species
TLC: Thin layer chromatography
V79 cells: Chinese hamster lung fibroblast cell lines
